# Allies in Orthopaedic Trauma Surgery

**DOI:** 10.1097/OI9.0000000000000098

**Published:** 2021-06-10

**Authors:** Lisa K. Cannada, Bradley Dart, Niloofar Dehghan, Kyle Jeray, Anna N. Miller

**Affiliations:** a Novant Health, Winston-Salem, NC; b Advanced Orthopaedic Associates, Wichita, KS; c The CORE Institute; d Department of Orthopaedic Surgery, University of Arizona College of Medicine Phoenix, Phoenix, AZ; e Department of Orthopaedic Surgery, University of South Carolina, Prisma Health, Greenville, SC; f Washington University Department of Orthopaedic Surgery, St. Louis, MO

**Keywords:** bullying, discrimination, diversity, sexual harassment

## Abstract

From the casting couch to the board room, the media, and beyond, the topic of sexual harassment and bullying can no longer be ignored. Sexual harassment and bullying in medicine has the potential to be the next big headline on these topics. The culture in medicine and especially the hierarchy in surgery often permit this behavior. To improve the culture in orthopaedic surgery regarding sexual harassment and bullying, education must occur. With education, comes acknowledgment and recognition. This permits an ability to act on and improve the culture. This paper will start the dialogue of this difficult topic and provide a call to action for sexual harassment and bullying to become “Never events”.

## Introduction

The topic of sexual harassment and bullying has been in the news for the past few years. Medicine has not yet made drastic headlines, but the problem exists. Some particularly eye-opening statistics include a report from the National Academy of Women in Science, Engineering and Medicine (NASEM) finding 50% of medical students reported being sexually harassed.^[1]^ In a work environment survey of the AAOS, 79% of respondents reported discrimination, 55% bullying, and 47% sexual harassment.^[2]^ Especially alarming was the repetitive nature of the behaviors and significant fear of reporting. The hierarchical structure of medicine may make one feel helpless if they witness or are the victim of these behaviors.

We hope to provide both education on the topic and tools for engagement, as well as to create a positive environment for all in orthopaedic trauma surgery. Our goal is to make what may have been previous accepted behavior a “never” event.

## Examining the culture

When faced with any challenge in society, it is best to first define the issues. While this may seem impossible, the more difficult task rests with answering the “why” or “how” leading to these challenges. With regard to harassment within surgery, it is important to examine our culture and values which define our society. Culture has been defined by Robert Kohls, the former director of the United States Information Agency, as an “integrated system of learned behavior patterns that are characteristic of members of any given society. Culture is the total way of life of particular groups of people. It includes everything that a group of people thinks, says, does and makes—its systems, attitudes and feelings. Culture is learned and transmitted from generation to generation.” While American culture displays many positive features which have led to a prosperous and innovative society, there are likely many other features we would rather not discuss.

When asked, most would have a difficult time coming to a consensus list regarding what constitutes “American Values”. Kohls described a list of 13 values present in America, a list that was designed to help foreign nationals understand more than 95% of American actions.^[3]^ Examining this list offers an introspective approach to gaps within our value system which may have led to the historical cultural acceptance of harassment within our society. It may also afford the tools necessary to further correct this dilemma. Of the 13 values Kohls described, the following will be examined: personal control, individualism, privacy, self-help, and competition.

First, orthopaedic surgeons, like most Americans, value “personal control” over their environment. It is the American way to refuse defeat. It is considered normal and right that man should control nature rather than the other way around. We view the mentality of fatalism as foolish and lazy. Why would we see the world any other way? After all, we put Americans on the moon, built the Model T, and invented the Internet. Controlling your own fate could hardly be interpreted as a negative value; however, it is possible this has driven immoral individuals to display a lack of deference for others.

Another value Americans share is their desire for individualism and privacy. We consider ourselves completely unique and totally different than all other individuals. Americans resist being part of a homogenous group. We value our differences and welcome varying perspectives. We tend to believe we are slightly different, even when faced with unacceptable behavior. This individualism can lead to excuses or a mentality that the rules do not apply. Certain individuals may find it difficult to accept and adopt ideas otherwise universally accepted. Conforming to rules with which one does not agree requires a greater sense of community and cooperation. Otherwise, one creates an environment where one individual is the rule maker and others are at a disadvantage.

Privacy, a symptom of the pure individual, is likely one of the most coveted values Americans hold. Even when the other virtues of our culture have failed, one will hold on to their right as an attempt to protect their personal control and individualism. Not only do we tend to protect our own privacy, we value others’ privacy as well. It is possible this value of privacy has kept many victims of harassment in the shadows and perpetuated the lack of awareness of the issue, thus creating a “culture of silence.” These 2 values likely drive victims and witnesses to keep to themselves. The individual must display great bravery to bring awareness to what is unknown to the group.

This dilemma is further complicated by the value of “self-help” which Americans also hold critical to personal accomplishment. Whether for reasons of privacy, embarrassment, or pride, we tend to attempt to fix our own issues. As the challenges associated with harassment and bullying are exposed, we may tend to hide our association with a situation as to avoid being seen as a victim or possibly an accomplice.

Competition challenges and forces the individual to produce the very best. It is this value Americans believe has allowed us to achieve some of the greatest accomplishments in history. It has also prevented us from fully recognizing the value of cooperation. It creates the prize and the cost. In certain individuals, the prize becomes conquering another individual with no regard for the cost. Even our public approach to dealing with harassment is competitive. Over the past several years, the number of public accusations has greatly increased. This is largely due to the bravery of many victims whom have been more vocal about their experiences. While the actions of the guilty are inexcusable, our competitive nature may force us to defeat the innocent at the risk of letting the guilty slip away.

While attempting to examine our “culture” through the perspective of our value system, we have uncovered possible links between what has made us a successful and progressive society and what may also create an environment susceptible to bullying and harassment. The world of orthopaedic surgery is not immune to this susceptible environment. We are highly competitive individuals with a high degree of “personal control,” often with a dose of arrogance, finding it difficult to ask for help. We, like many other professional environments, are susceptible to the negative forces within society leading to bullying and harassment.

Fortunately, we are changing. The values described above are not to be any less desired. We must focus on the virtuous nature of these values to create a more acceptable environment. Change in the American mind is linked to development, improvement, progress, and growth. Our desire for change, especially in light of grievances, is considered a necessary American value.

In addition to our desire for change, we hold that all are created equally. In general, most Americans value equality. However, in medicine, we must contend with the system of rank and status, especially in the world of medical education.

Our future is what we hold most important. We must develop an environment where all members of the hierarchy are treated with equal respect and dignity. No longer can we afford to excuse the abuses of the past as “part of the experience” or “harmless.” We can and need to do this. It is inherent to our values.

## The culture in surgery

Let's turn to Australia. On Friday night, March 6, 2015, Dr. Gabrielle McMullin was completing a book signing. She is a vascular surgeon, and she commented on the case of Dr. Carolyn Tan. Dr. Tan won $100,000.00 in a tribunal hearing against her Neurosurgery attending for sexual harassment. However, since that occurred, she has been unable to find a job in a public hospital in Australia. Dr. McMullin suggested that Dr. Tan would have been better off giving in to the request for sexual favors. Concurrently, a conference of senior female executives in Australia said women returning to work after having children should ensure they use adequate birth control and that work-life balance really did not exist. As one can imagine, a media storm ensued. The reaction to Dr. Gabrielle McMullin was swift, with comments by senior medical personnel and multiple articles describing the widespread extent of the problem.

In Australia, all surgical specialties fall under the Royal Australian College of Surgeons. With the fallout of these incidents, the Royal Australian College of Surgeons stated that clear guidelines against harassment in the profession were needed, and, shortly thereafter, established an Expert Advisory Group (EAG), mounted an improved complaints process, and partnered with an independent external agency to provide a support program for those affected. The goal was to obtain information on the extent of discrimination, bullying, and sexual harassment, known as DBSH.

The EAG surveys were helpful to define the extent of the problem. One half of all the members reported in the survey that they have been victims of DBSH including 63% of trainees and 30% of women—but the problem was there regardless of gender.^[4]^ Hospitals were asked and 71% noted this behavior by surgeons.^[4]^ (As an aside, we generally do not ask hospitals in the US about physician behaviors.) Bullying was the most prevalent form of behavior and male consultants were most often the perpetrators. Oftentimes, the behavior occurred on multiple occasions. In more than half of cases, it occurred on more than 3 occasions and continued even if reported. Up to 20% of respondents left their position.

In late 2015, the EAG came out with 42 recommendations to be followed over a 5-year period. The action plan emphasized the following: change the culture and leadership; education of all; the development of a diversity and inclusion plan to have more women in leadership positions; creation of a complaints management process.^[5]^

By the end of 2017, license renewal in Australia could not occur without compliance with the education program. There was mandated completion of training modules and additional training for those involved in teaching residents.

## The culture in medicine in the United States

From the casting couch to the board room to television and the athletic field, the harassment of females is finally being taken seriously in the United States. In the medical field, people are also starting to take notice of these issues (Table 1).

**Table 1:** Examples of medicine in the headlines

***October 2017*** University of Southern California (USC) lost its second Dean for misconduct after reports surfaced on a 2003 discipline for harassment.
***February 2018*** NBC news broached the topic covering the surgeons at Loma Linda University.
***November 2018*** University of Maryland made the headlines, and more importantly the surgeon involved was able to move to a more prestigious institution, despite the claims of harassment.
***November 2018*** A news investigation found Saint Louis University is a hostile environment where medical students and trainees feared retaliation if they complained and an environment where discriminatory behaviors were occurring on a regular basis. The ACGME completed a site wide visit due to reports of retaliation.
***December 2018*** At Yale, there was promotion/recognition of a professor with claims of sexual harassment. The Dean ultimately resigned.

Why does this behavior exist in medicine? Medicine is a very hierarchical field. Consider the hierarchy within most departments. Medical students report to interns, interns to senior residents, residents to faculty, faculty to department chairs, chairs to deans, etc. The power gap increases with each step. However, the competitive forces are inverted within this pyramid creating a prime environment where highly competitive individuals are subordinate and susceptible to predators.

Also, power is concentrated in certain individuals, those who train you and assess you. Think about the leaders in surgery, including orthopaedics with 94% of AAOS members being male.^[6]^ In surgery there is a ladder and power differential which permits bullying behavior. When an event happens, reporting is discouraged. The majority of events are most likely unreported, as there is often significant fear of retaliation and damage to one's career.

The behavior that is reported in the literature affects everyone, from medical students to full professors to hospital personnel, and includes psychological abuse, repeated bullying and retaliation, sexual harassment, and even physical abuse. We should have ZERO tolerance for this behavior.

As physicians we have high standards from society. We are in a position of prestige and have been given the privilege to preserve human life and function. We are expected to be role models of ethical and respectful behavior. How we behave shapes our culture and profession. In addition, we need to create a respectful environment which improves patient safety. It has been proven that trainees and others are afraid to discuss complications of physicians who bully them, so patient care can be affected.

Let's look more closely at gender bias in medicine. Many examples exist, including salary disparity, women lacking in leadership roles, females reporting less job satisfaction and being subject to intimidating behavior. Gender bias is often encountered by women if they are self-confident, successful, and driven. Ambition is thought to be a less desired trait for women in general, and this applies to medicine as well.

Why do women put up with it? Women get used to it, becoming desensitized. Women are driven by the goal of caring for their patients. The hierarchy continues to exist and, should one desire to change something, it often is too much hassle.

But DBSH is not just due to gender. Affected individuals include those with cultural, ethnic, or racial differences; disabled individuals, those with alternative lifestyles, different religious beliefs, and aging physicians.

It is important to remember that no one asks to be a victim and, until you experience it, you cannot begin to understand the profound effect on one's personal, professional, and family life. We should not listen to what others do, we should lead by example. We can evaluate the problem by obtaining data, similar to the action taken in Australia. We should develop resources, including the ability to report without fear and actual consequences for those involved. Medicine can lead by example. A diversity plan to improve the culture in medicine should be a part of every specialty and should be in the publication of articles, awarding of grants, roles in committees, and leadership positions. Nowhere do women want to be put in leadership roles due to quotas, but rather want to be in their roles because they deserve it, and not excluded from them due to their gender or other diverse attributes. And, speaking of leadership roles, perhaps every application for committee members, chairs, Board of Director's positions should include: Have you had a finding against you of sexual harassment or discriminatory behavior?

## Improving the culture

### Starts at the top

One of the issues with discrimination is that it is a result of unconscious bias, which is so prevalent in society. A person's unconscious bias can cause them to have certain beliefs regarding those of different gender/religion/race/class, and treat them differently without intending to do so. Addressing discrimination starts with changing this unconscious bias, by changing the culture and social norms. While changing social norms in society can take time and is difficult to enforce, changing the culture in the work place is in our control. Creating an equitable, safe, and collegial work place can create an environment where differences are valued, and allows for better patient care.

Changing the culture at the work place starts from the top. For example: Do you think a woman could be an effective president of the United States? Or the OTA? Would that even be a question if 50% of past presidents were female? Likely not. Given that, one has to stop and actively visualize this unusual scenario to make it more acceptable.

While the majority of medical students are female, as one advances in training and residency in orthopaedics and examines leadership positions, the majority are male.^[7]^ Cultural and racial minorities are also few and far between when it comes to higher positions in medicine, such as department chair or dean. If people of visible minorities are routinely seen in positions of leadership, it helps change the social norms and expectations, and helps eliminate the unconscious thought questioning their ability to lead.

Leaders represent the culture of the organization they work for. Hence, the search for a leader should include questions about their values, beliefs, past behavior, and any issues of discrimination, bullying, or sexual harassment.

Leaders can also create and foster a discrimination-free environment by example; by the way they behave, the people they recruit, and the policies they enforce. Leaders have a huge responsibility in shaping the culture of their work environment, and should educate and stop any prejudicial behavior. For example, if an inappropriate joke is cited during morning rounds, and the department chair laughs (or does nothing), it creates an environment where discriminatory behavior is tolerated and promoted. In addition, education of bystanders for “bystander intervention” is something to strongly consider (Table 2). The ultimate goal is to stop the behavior in its tracks, and provide support for individuals being harassed.

**Table 2:** Bystander intervention: the concept of taking attention away from the person being harassed

***Direct*** – use verbal or nonverbal clues to let the person know that the behavior is not acceptable; ask them questions loudly
***Distract*** – change the topic, trip, spill a drink, comment on the person being targeted and their accomplishments
***Delegate*** – tell somebody who is in authority
***Delay*** – support the victim after witnessing the event: “I am sorry for what happened”
***Document*** – written documentation of event
More information: https://www.traliant.com/bystander-intervention-training

### Policies and supporting action

Additional ways we can help improve social norms in the work place are by taking explicit steps to recruit a diverse faculty at every level. To recruit diversity, the recruitment committee also needs to be compromised of a diverse group.

It can be difficult to promote minorities, without making a conscious effort to do so, and creating specific policies to support them. For example, if the leadership of a national meeting banned presentations by all-male panels of speakers (the so called Manels), as the leader of the National Institutes of Health has done, it would create more opportunities for women in such positions, normalize their presence, and hence open the door for future generations. This is also true for minorities. If there are specific policies in the work place or organization to recruit, promote, or retain people of minority groups, this will help foster a more diverse environment.

There can be barriers toward recruitment and retention of minority groups within an organization, which need to be identified and addressed. For example: “I am an orthopaedic surgeon, and a new mother. I need to pump breast milk every 3-4 hours to be able to feed my baby. How do I do this during a busy surgery or clinic day without having it affect my work and productivity? How do I get my employer or partners to understand and not view it as a negative side of being a female surgeon?” The Accreditation Council for Graduate Medical Education (ACGME) recognized the importance of starting in residency education with policies. In July 2019, the ACGME Common Program Requirements mandated that residency and fellowship training sites must provide private and clean locations where residents may lactate and store the milk within a refrigerator [which] should be in close proximity to clinical responsibilities. It would be helpful to have additional support within these locations that may assist the resident with the continued care of patients, such as a computer and a phone, as the time required for lactation is also critical for the well-being of the resident and the resident's family.^[8]^ Hopefully this will spread to consideration for all hospitals and practices for female surgeons. If the culture we work in supported women's maternity leave or new mothers’ needs, there would not be such an anxiety amongst these surgeons about how they will be treated once they have a baby. In fact, we should regard this as parental leave, and both men and women should partake and participate.

Establishing mentoring networks is also beneficial, and institutional or national societies for minorities can help with networking and mentorship opportunities. This helps create a safe haven for people to connect with others who have similar experiences, and find support and solutions.

## What exists currently

Since the adoption of the nineteenth amendment, which prohibited denying the right to vote on the basis of sex, organizations have striven to improve equality at all levels. Nationally, the most commonly recognized example of this (now commonly known as “title IX”) was enacted in 1972 by Richard Nixon. This specifically prohibits organizations and programs from receiving federal funds if they discriminate between sexes. This was fully enacted and enforced by 1980 with financial penalties in place. The United States Department of Education's Office for Civil Rights is the enforcing body for title IX, and this includes its policies that cover sexual harassment in addition to discrimination. The other main governing body enforcing workplace equality is the United States Equal Employment Opportunity Commission (EEOC), which was established in 1965, and stems from a mandate in title VII of the Civil Rights Act. The EEOC also considers discrimination against sexual orientation to be included. More recently, the Notification and Federal Employee Antidiscrimination and Retaliation Act (No FEAR) was instituted to improve federal employees’ ability to report discrimination without retaliation and understand their rights regarding reporting.

As discussed previously, medicine, and particularly orthopaedic surgery, is a field that may be fraught with concern for sexual discrimination and harassment; therefore, many governing bodies have already worked to write policies regarding these issues. The American College of Surgeons states that there should be zero tolerance for harassment and that surgical training curricula should include a focus on these areas.^[9]^ The Eastern Association for Surgeons in Trauma (EAST) has provided guidelines as a result of their task force.^[10]^ The American Medical Association discusses a zero tolerance policy and that participants should always “exercise consideration and respect” for those around them.^[11]^ The Accreditation Council for Graduate Medical Education includes a harassment section in their Institutional Requirements stating that residents and fellows should be able to “raise and resolve.” Within our own orthopaedic leadership, the American Academy of Orthopaedic Surgeons “offers aspirational advice on how an orthopaedic surgeon can best deal with a particular situation.”^[12]^ This document specifically notes that, “Perceptions of what constitutes offensive behavior sometimes differ between men and women.” In addition, “Orthopaedic surgeons should ensure that their actions cannot be considered sexual harassment even by the most critical observer.” At this time, no major orthopaedic subspecialty organizations have their own sexual harassment policies.

## Call to action

The topic of sexual harassment, discrimination, and bullying has garnered some attention in orthopaedic surgery, yet the data clearly shows these problems continue to exist in our profession. The goal of white papers like this is to help increase awareness, establish best practices, and provide resources and toolkits to deal with these issues and others. Many organizations and institutes are working to address these issues, often with policies and procedures directed at unacceptable behavior. But ultimately it is the institution and workers that can address these topics (and fix them) and minimize or eliminate their impact in the environment. Leaders in our field have the opportunity to influence the culture in their respective departments and should not tolerate unprofessional behaviors. By setting standards, the culture can change. Many universities are stepping up and launching initiatives aimed at preventing sexual harassment, and creating a culture of safety, respect, and equity. Ongoing research is important to help continue to define the extent of the problems and help provide solutions.

Below is a short list outlining a call to action:

1. Acknowledgement that these issues are a problem in orthopaedic surgery
2. Zero-tolerance policies
3. Transparent systems for monitoring unacceptable behaviors
4. Transparent, safe, and anonymous reporting systems (without fear of retaliation)
5. Mandatory training/education
6. Mentorship, dialogue, and collaboration
7. Continued assessment and research
8. Continued grass-roots efforts that will ultimately drive local, regional, and national policies

By embracing these efforts, the goal is to create a cultural shift where medical professionals feel empowered to call out unacceptable behaviors and microaggressions, whether it be toward them or others. And training will help make this happen. Movements such as the #MeToo have helped change the mantra that those “at the top” cannot be stopped or changed. But with solidarity, each of us can find our voice, feel protected, and change the culture, fostering a more inclusive learning and workplace environment.

In closing, we ask that we create a culture of caring. It is important that as physicians we are aware, care, and act for the best interests of the profession to promote gender equity and to improve the culture of medicine.

This call to action is ultimately for all health care professionals to open our minds, challenge ourselves to identify and combat our biases, and welcome everyone to the table as a collective of equals. Our specialty, our peers, and the patients whom we treat, deserve this.
